# The role of stochasticity in fungal community assembly: explaining apparent stochasticity with field experiments

**DOI:** 10.1098/rspb.2024.2416

**Published:** 2025-02-05

**Authors:** Nerea Abrego, Sonja Saine, Reijo Penttilä, Brendan Furneaux, Tuija Hytönen, Otto Miettinen, Norman Monkhouse, Raisa Mäkipää, Jorma Pennanen, Evgeny V. Zakharov, Otso Ovaskainen

**Affiliations:** ^1^ Department of Biological and Environmental Science, University of Jyväskylä, PO Box 35, Jyväskylä FI-40014, Finland; ^2^ Department of Agricultural Sciences, University of Helsinki, PO Box 27, Helsinki FI-00014, Finland; ^3^ Natural Resources Institute Finland (LUKE), Helsinki 00790, Finland; ^4^ Finnish Museum of Natural History, University of Helsinki, PO Box 7, Helsinki FI-00014, Finland; ^5^ The Canadian Centre for DNA Barcoding, Centre for Biodiversity Genomics, University of Guelph, Guelph, Ontario, Canada, N1G 2W1; ^6^ Department of Integrative Biology, College of Biological Sciences, University of Guelph, Guelph, Ontario, Canada, N1G 2W1; ^7^ Organismal and Evolutionary Biology Research Programme, Faculty of Biological and Environmental Sciences, University of Helsinki, PO Box 65, Helsinki FI-00014, Finland

**Keywords:** colonization, community assembly, community divergence, ecological drift, succession, wood-inhabiting fungi

## Abstract

Stochasticity is a main process in community assembly. However, experimental studies rarely target stochasticity in natural communities, and hence experimental validation of stochasticity estimates in observational studies is lacking. Here, we combine experimental and observational data to unravel the role of stochasticity in the assembly of wood-inhabiting fungi. We carried out a replicated field experiment where the natural colonization of a focal fungal species was simulated through inoculation, and the local fungal communities were monitored through DNA metabarcoding before and after the inoculations. The amount of stochasticity in fungal colonization was less pronounced than expected from the amount of unpredictability in observational data, suggesting that stochasticity may play a smaller role in fungal occurrence than previously anticipated, or that it may be a stronger influence in the dispersal and establishment phases than in colonization *per se*. Stochasticity was more prominent in the initial phase of community succession, with the earliest successional stage involving a higher level of stochasticity than the later stage after 2 years. We conclude that experimentally measuring the role of stochasticity in community assembly is feasible for species-rich communities under natural conditions and highlight the importance of experimentally testing the accuracy of stochasticity estimates based on observational data.

## 1. Introduction

Stochasticity is one of the principal processes of community assembly, and it is well known to play a central role in determining the composition of ecological communities [1,2]. Stochastic processes generate divergence even among communities occupying identical environments. In a community, as in a population, two main forms of ecological stochasticity operate simultaneously: stochasticity caused by intrinsic demographic factors (e.g. births, deaths, migrations) and stochasticity caused by extrinsic environmental factors (e.g. unpredictable weather events). How these two forms of stochasticity jointly operate at the level of populations has long been studied theoretically as well as tested empirically [3]. More recently, stochasticity has been explicitly incorporated in frameworks of community assembly [1,2], and several empirical studies have demonstrated the importance of stochasticity in structuring communities [4,5]. However, measuring the true contribution of stochasticity from empirical systems has proved challenging, especially for species-rich communities, as it is difficult to disentangle stochasticity from confounding factors.

At the level of communities, the effects of stochasticity propagate across species and additionally, stochasticity interacts with other ecological processes, such as abiotic environmental selection [6–8] and interspecific interactions [9,10]. Directly measuring how exactly stochasticity operates in species-rich systems may thus be challenging, as monitoring the population dynamics of each of the constituent species is often not feasible, nor is knowing how the species within the local community interact. As a result, stochasticity is often measured using indirect methods that aim to capture the signatures of stochasticity from community patterns [11–13]. For example, null-model approaches have been designed to detect and quantify the community variance that cannot be attributed to the environmental factors controlled in the experiments or in the field observations. Such methods are often applied to non-manipulative data of species-rich systems [13,14], in particular, those of microbial communities [15–18].

Much research on the role of stochasticity has focused on species’ succession, assessing whether community trajectories are predictable over time [2,18]. It has been shown that the stochastic processes are most relevant in the initial phases of succession, and in the latter phases, communities become more predictable [19,20]. Such temporal dynamics is closely related to the influence of priority effects, i.e. the effects of the preceding biotic and abiotic environments on the community pathways [21]. Because it is often difficult to measure exactly when a given individual migrates into the local community and how this modifies the biotic and abiotic environments, the influence of priority effects may appear as stochastic variation [6,22]. Furthermore, while the timing of priority effects (e.g. the exact timing when a given individual with a given identity migrates into the local community) may be stochastic, priority effects (i.e. the influence of such migration in the biotic and abiotic environments) themselves may be predictable.

Owing to the difficulties in directly measuring stochasticity, it has often been assumed that the level of stochasticity is reflected in the amount of unpredictability in a focal community. Yet, in addition to true stochasticity, unpredictability can relate to our inability to understand community dynamics. In fact, many factors other than stochasticity can produce unpredictability, importantly those which we fail to incorporate in the analyses simply because community dynamics are highly complex and *a priori* unknown. While the most relevant environmental abiotic predictors influencing a focal community are usually known, we often lack information on how species and individuals interact with each other or how and when migration events occur. Furthermore, ecological stochasticity may also be confounded with observational error or imperfect data collection [2]. Unpredictable community patterns may thus reflect true stochasticity and/or uncontrolled ecological processes and observational error. We refer to the unpredictability that is not owing to true stochasticity as apparent stochasticity.

**Figure 1.**
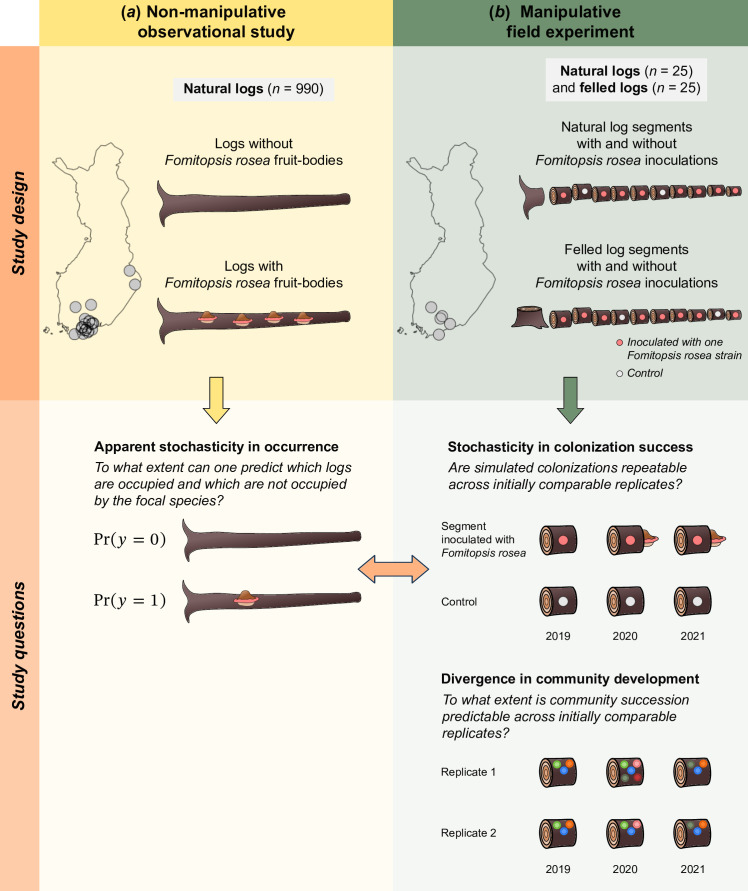
A conceptual presentation of the study design and study questions. The observational study (*a*) was used to measure the amount of apparent stochasticity in fungal occurrences. The manipulative colonization experiment (*b*) was designed to dissect the amount of true stochasticity in one critical phase of the species lifecycle, namely, resource acquisition and fruit-body production after dispersal and initial establishment. The figure illustrates how inoculated logs were predicted to become colonized by the target species, with the uncolonized control logs verifying the causality of the inoculations. The comparison between the observational field study and the manipulative colonization experiment enabled us to evaluate to what extent the colonization phase can explain the high level of unpredictability in fungal occurrences. In addition to the single-species study of *Fomitopsis rosea*, we used the same experimental units to evaluate the amount of stochastic divergence in the entire resident community. The figure illustrates the expectation that the initial phase experiences more stochasticity than the later phase of community succession (experimental units that are initially similar in 2019 diverge in 2020 but then converge again in 2021).

In this study, we combined data from a manipulative field experiment with non-manipulative observational data to unravel the role of stochasticity in structuring wood-inhabiting fungal communities (figure 1). The experiment included 500 replicates of local wood-inhabiting fungal communities. As replicates, we used segments of the logs, which we conceptually considered as habitat patches with as similar biotic and abiotic conditions as possible under natural conditions. Within the replicates, we inoculated different strains of a focal fungal species, the rose bracket fungus *Fomitopsis rosea* (syn. *Rhodofomes rouseus*). We considered the inoculations of different strains as simulated natural colonization events of different individuals of the same species. Additionally, to include variation in the abiotic and biotic conditions of the habitat patches, half of the replicates belonged to logs that had recently naturally fallen, and half to logs that were artificially created by felling. The colonization success of the inoculated species and the development of the resident community were monitored by DNA-based surveys once before the inoculations and annually for 2 years after the inoculations. After 2 years, we additionally monitored the colonization success of *F. rosea* with a fruit-body-based survey. The non-manipulative observational data consisted of 39 observations of *F. rosea* under 990 sampling units that were comparable with those used in the experiment.

We expected to find a level of divergence in community succession between the comparable replicates as well as a degree of stochasticity in the colonization success of *F. rosea*, but to a lesser extent than estimated from the non-manipulative observational data. This is because with a high number of similar habitat patch replicates, it is possible to quantify the roles of biotic and abiotic conditions on community assembly, and thus explain part of the apparent stochasticity recorded in non-manipulative data. Non-manipulative data of wood-inhabiting fungal communities involve high variation in both species communities and environmental conditions, which are difficult to rigorously disentangle. Likewise, we expected the inoculation experiment of *F. rosea* to show the importance of the colonizing individual, an effect not possible to measure in non-manipulative studies. Furthermore, following the study of Chase [6], we expected to find an interaction between stochasticity and environmental selection: we hypothesized that the role of stochasticity would be larger in naturally fallen dead wood units than in felled units, as natural units hold more diverse fungal communities [23] and thus, higher biotic complexity. In line with earlier findings in other systems [19,20], we expected to find a larger divergence among local communities in the early phase of the experiment than in the later stage. Finally, we expected different survey methods to yield qualitatively consistent but quantitatively different results, because DNA metabarcoding is prone to false negatives and positives, whereas morphological observations are especially prone to false negatives [24].

## 2. Material and methods

### (a) Experimental study area and experimental design

We implemented the experiment in five forest sites located in southern and central Finland, the same as in Saine *et al*. [23] (electronic supplementary material, appendix 1). Briefly, the study sites were located in protected areas or set-aside forests that were spruce-dominated, with a middle-aged or mature stand age and a high amount of deadwood (electronic supplementary material, appendix 1 table S1). The size of the study sites varied between 2 and 5 ha (electronic supplementary material, appendix 1 table S1).

The experimental units comprised five hundred 1 m Norway spruce (*Picea abies* (L.) Karst) log segments that originated from 50 logs in five forest sites (10 logs per site, 10 replicates per log) in southern and central Finland. Out of the 50 logs, 25 were naturally fallen and 25 felled with a chainsaw. The experiment was initiated in April–May 2019. In each site, we selected five natural logs and felled five living spruces. All logs had a diameter at breast height ≥ 20 cm. The natural logs were selected to be as freshly fallen as possible (we targeted decay class 1 but also selected 2 if 1 was not available). For the felled logs, we selected healthy living spruces and cut them from the base at approximately 25 cm using a chainsaw. To obtain replicates representing as similar biotic and abiotic conditions as possible, we cut each log into ten 1 m-long segments using a chainsaw. Neither the chainsaw nor the segment surfaces were sterilized. The first segment was located at the base of the log. To avoid direct contact among log segments and ensure independent development of fungal communities, the segments were slightly moved after cutting so that they were separated by approximately 5−10 cm. A summary of the abiotic segment characteristics (electronic supplementary material, appendix 1 table S2) and the initial fungal community composition (electronic supplementary material, appendix 1 table S1) is presented in the supplements.

As segment-level environmental characteristics, we recorded the diameter in the middle of the segment (cm), decay class (1 or 2 following Renvall’s [25] classification) and the proportion of the ground contact and bark cover (0−100% in intervals of 10%). All characteristics were measured at the start of the experiment in 2019, and the decay class was remeasured at the end of the experiment in 2021. At the log level, we recorded log type (natural or felled) and mortality factor for natural logs (broken or uprooted). The average diameter of the first segment was 28.8 ± 4.5 cm for natural logs and 28.6 ± 4.1 cm for felled logs. Out of the natural logs, 68% were broken and 32% uprooted. At the beginning of the experiment in 2019, all 250 segments of felled logs were in decay class 1 and out of the 250 segments of natural logs, 67.2% were in decay class 1, 32.4% in decay class 2 and 0.4% in decay class 3. At the end of the experiment 2 years later, all felled segments remained in decay class 1, while 14% of natural segments had progressed to a more advanced decay class, with 54.4% of natural segments in decay class 1, 42.8% in decay class 2 and 2.8% in decay class 3.

### (b) Inoculations of *Fomitopsis rosea*

*Fomitopsis rosea* was selected as the focal species because it is naturally rare or absent in the study sites, and thus new fruiting bodies could be considered to originate from the inoculations. Moreover, *F. rosea* is an easy species to recognize in the field and easy to culture in the laboratory. From an ecological point of view, *F. rosea* is a habitat specialist species, occurring mostly on intermediately decayed large spruce logs in old-growth forests, and it is included in the Red List of Finnish Species classified as near threatened [26]. We employed eight strains of the focal fungal species *F. rosea* for the inoculations. The collection of source material, mycelial cultivation and dowel preparation followed the workflow described in full detail in the electronic supplementary material, appendix 2. To summarize, in August–November 2018, *F. rosea* fruit bodies were collected from eight natural populations in different locations in Finland (electronic supplementary material, appendix 2 table S3, figure S2). We then established fungal cultures from the source material at the Natural Resources Institute Finland (LUKE) by growing the strains on agar plates for 19−54 days, depending on the strain (electronic supplementary material, appendix 2 table S3, text S1). The identifications of *F. rosea* were confirmed by applying Sanger sequencing to each strain (electronic supplementary material, appendix 2 text S1). At Kääpä Biotech Oy (Karjalohja, Finland), mycelia were moved to 50 × 10 mm sterilized wooden dowels made of industrial spruce timber (Helsingin Erikoishöyläys Oy, Finland) by placing both the mycelia and the dowels in plastic grow bags filled with oat grains, which helped the strains to colonize the dowels.

The inoculations were carried out in August–October 2019 by inserting colonized dowels in each log segment. Within the 10 segments of each log, we inoculated a different *F. rosea* strain in each of the eight segments and uninoculated control dowels with no mycelia in the remaining two segments, in a randomized order. For this, we drilled three holes in a triangular shape (approx. 3 cm apart) on the side of each segment using a cordless drill (Makita, model DDF481) and a wood drill bit (11 × 105 mm) and introduced two dowels in each hole. To prevent contamination, we covered the holes with gardening wax (Oy Neko Ab, Finland). The drill bit was sterilized after each segment by soaking it in sodium hypochlorite (5% NaClO) for at least 3 min, then washed with normal water and finally with ethanol. Each of the eight strains was inoculated in 10 segments per site and thus, in 50 segments in total.

Before the inoculations, we checked that *F. rosea* fruit bodies did not naturally occur in the segments. Additionally, two fungal experts conducted an 8 h fruit-body survey at each site in autumn 2019, surveying all logs within the study sites as well as their immediate surroundings. Fruit bodies of *F. rosea* were recorded in a total of nine non-focal logs in the surroundings of three sites. However, the inoculations were carried out as planned since the occurrence rates were low, and the target species did not occur within the study sites.

### (c) Monitoring of colonization success and resident community succession

We followed the colonization success of *F. rosea* as well as the development of resident fungal community composition by collecting sawdust samples from the log segments and applying DNA metabarcoding. Altogether, we collected three sets of sawdust samples. The first set of samples was collected before the inoculations. We collected these samples at the same time as the inoculations in August–October 2019, by gathering the sawdust into plastic zip lock bags (which were assumed to be free of wood-inhabiting fungal DNA) from the holes that were drilled for the subsequent inoculations. To follow the colonization success of *F. rosea* and the succession of resident communities after the inoculations, we collected the second and third sets of sawdust samples 1 and 2 years after the inoculations, in August–September 2020 and 2021, respectively. We drilled holes approximately 2 cm away from each inoculation point with a cordless drill and a metal drill bit (6 × 93 mm, Bosch, product code 2608585926) and collected the sawdust in plastic zip lock bags. Drill bits were sterilized between uses with sodium hypochlorite as explained above. The bark was removed to ensure targeting fungi inhabiting the wood. Two years after the inoculations in 2021, we additionally surveyed the segments for *F. rosea* fruit bodies to obtain morphological records of the colonization success.

### (d) DNA metabarcoding and bioinformatic analyses

Workflow for the sample pre-processing, DNA extraction, DNA amplification using polymerase chain reaction (PCR), sequencing and bioinformatics followed Saine *et al*. [23]. More details on each step are in the electronic supplementary material, appendix 3. After field collection, the samples were stored at −20°C as sawdust in sample tubes for a period of 5−45 weeks before freeze-drying. The sawdust samples were then pulverized with a homogenizer in preparation for DNA sequencing. For the grinding, we placed a sample and sterile metal beads (6 × 4 mm and 2 × 10 mm) in a metallic grinding jar (25 or 50 ml) with sterile tweezers and ran a homogenizer (Mixer Mill MM 400, Retsch) for 5 min at a 30 s^−1^ frequency. After each sample, we cleaned the grinding jars by rinsing with water, drying, treating with a DNA/RNA decontamination solution (PDS-250, Biosan SIA) and wiping them with clean paper towels and dry-heated the metal beads and tweezers after washing them for 4 h at 200°C.

The PCR amplifications targeted the ITS2 region (internal transcribed spacer 2) using primers ITS3 and ITS4 [27]. A mix of nine synthetic sequences flanked by the ITS3 and ITS4 primer sites, based on those of Palmer *et al.* [28], was added to the PCR master mix as a spike-in to allow quantification of DNA amount at the per-sample level (as applied in Ovaskainen *et al*. [29]). Paired-end 2 × 300 bp sequencing was performed using an Illumina MiSeq instrument with 84 samples plus one DNA extraction negative control and two PCR-negative controls per sequencing run. Because the spike-in sequences were added to the PCR master mix, these were also present in the extraction and PCR-negative controls. Four of the sequencing runs also included nine negative sequencing controls, which did not include even spike sequences. A total of 28 sequencing runs were included in the study. In the bioinformatic pipeline, raw sequences were trimmed, filtered and denoised using DADA2 with default parameters and merged to form amplicon sequence variants (ASVs). Taxonomic identification of the ASVs was done using probabilistic taxonomic placement with Protax-Fungi [30], applying a 50% probability threshold [31]. Sanger sequences from the strains used in the study were added to the Protax-Fungi reference data in order to ensure success in identifying those strains in particular. ASVs were clustered using a taxonomically informed clustering approach to form operational taxonomic units (OTUs) that were used at the species rank for the ecological analyses.

Sequencing generated a mean of 227 621 raw read pairs per sample (min: 747, max: 1 218 335), excluding the negative sequencing controls. A mean of 88 206 read pairs (min: 20, max: 442 537) passed all processing stages through spike detection. Study samples contained a mean of 53 434 (min: 0, max: 422427) non-spike fungal reads, while negative controls contained a mean of 148 (min: 0, max: 2054). Of the 84 extractions and PCR-negative controls, non-spike fungal sequences were present in 25. Negative sequencing controls generated a mean of 66 raw read pairs (min: 17, max: 186), of which a mean of 0.3 (min: 0, max: 5) passed processing stages through spike detection and a mean of 0.1 (min: 0, max: 2) were identified as non-spike fungal reads. The OTUs detected were less abundant in the negative controls where they occurred than in the mean of other samples, indicating that their presence in the negative controls was owing to low-level cross-contamination (either laboratory or bioinformatic) rather than systematic contamination. Study samples contained a mean of 37.7 fungal OTUs (min: 1, max: 240).

### (e) Observational study area and study design

We compiled data from earlier fungal surveys that were conducted in spruce-dominated forests in southern, central and eastern Finland (electronic supplementary material, appendix 4). We selected surveys that had recorded log-level data, and that included *F. rosea* in the list of target species. To make the results comparable, we considered observational data representing similar environmental conditions as in the experiment. We selected only large (>14 cm in diameter) spruce logs in decay classes 2 or 3. This resulted in data originating from 86 study areas, including a total of 990 surveyed logs, out of which *F. rosea* fruit bodies were detected in 39. For each log, the data contained information about the log mortality factor (*n* = 255 for broken and *n* = 735 uprooted), diameter (min: 14 cm, max: 67 cm and mean: 25.2) and decay class (*n* = 504 for 2 and *n* = 486 for 3) (electronic supplementary material, appendix 4 table S4).

### (f) Statistical analyses

As the main analytical approach, we used generalized linear mixed modelling, implemented through the joint species distribution model of hierarchical modelling of species communities (HMSC) [32,33]. This approach allowed us to measure predictability in the occurrence and composition of fungal communities in relation to the environmental predictors, while also accounting for the spatial and temporal structure of the data. To answer our main study questions, we fitted three sets of models. First, we used the experimental data to unravel the true role of stochasticity in the colonization success of *F. rosea*. Second, we measured the predictability of the same species from non-manipulative data on species occurrences. Third, we measured the stochastic component of community succession across the replicates originating from the same log.

#### (i) How unpredictable is colonization success and what fraction of it is true stochasticity?

To measure to what extent colonization success is unpredictable, we included in the analyses those 400 log segments inoculated with *F. rosea*. The 100 control segments were used to confirm that the colonization success of *F. rosea* in the inoculated segments could be reliably attributed to the treatment. We measured the predictability of colonization success in the following three ways: (i) fruit-body presence–absence, where we considered colonization by *F. rosea* to be successful if its fruit body was detected 2 years after the inoculation; (ii) DNA presence–absence, where we considered colonization by *F. rosea* to be successful if its sequences were detected either 1 or 2 years after inoculation; and (iii) DNA abundance conditional on presence, where the number of sequence reads classified as *F. rosea* was summed over the post-inoculation years, with zeros masked as NA, and then the values log-transformed and scaled to zero mean and unit variance. We incorporated these three response variables in a tri-variate HMSC model, assuming probit link function and Bernoulli distribution for the presence–absence response variables, and normal distribution for abundance conditional on presence. As fixed effects in the models, we included variables representing abiotic environmental conditions (log mortality factor, as broken/uprooted/felled, and diameter, decay class, ground contact and bark cover of the segment); variables representing the biotic conditions based on the resident community data from 2019 before the inoculations (DNA amount, OTU richness and community structure measured by the first four latent variables of a gllvm-model [34] fitted to presence–absence data); a variable representing the identity of the colonizing individual (fungal strain) and a variable representing sampling effort (log-transformed number of sequence reads). As random effects, we included the log and site identities, with the motivation that these capture latent log- and site-level covariates that have influenced all segments of the same log in the same way. We measured predictability by Tjur’s *R*^2^ [35] for presence–absence and classical *R*^2^ for abundance conditional on presence, both based on 10-fold cross-validation and defined the amount of apparent stochasticity as one minus predictability. To quantify the role of each of the variables in explaining colonization success, we applied a variance partitioning approach [36]. To measure the level of stochasticity separately for naturally occurring and felled logs, we also repeated the analysis separately for these two subsets of 200 log segments. While naturally fallen logs are more comparable with the logs in observational data, we note that fungi can already colonize logs before they fall down or very soon after that [23] and much before producing fruit bodies [37]. Hence, the felled logs may mimic more closely those natural logs that the species encounters in the colonization phase.

#### (ii) How predictable are *Fomitopsis rosea* occurrences in the observational data?

To quantify the amount of apparent stochasticity from observational data, we fitted a univariate HMSC model to the observational data, where we applied probit regression to model the presence–absence of *F. rosea*. As fixed effects, we included log mortality factor (broken/uprooted), diameter and decay class. We fitted two variants of the model, where we either included or excluded the study area as a random effect. The motivation for considering these two model variants is that the study area is likely to influence the species occurrences through multiple processes (e.g. colonization–extinction stochasticity at the level of study areas, proximity to source populations, microclimatic conditions), the effects of which are difficult to causally disentangle in such an observational study. Thus, the model with the random effect is likely to yield a higher predictive power, yet leaving it open to which processes or factors influence the species’ occurrences. We defined the amount of apparent stochasticity as one minus predictability and measured predictability by Tjur’s *R*^2^ based on 10-fold cross-validation. To evaluate to what extent different predictors contributed to the model, we partitioned the explained variation among the predictors.

#### (iii) Divergence in community composition during succession

We measured the divergence in community development in terms of four facets of community structure: fungal DNA amount, OTU richness and the first two latent variables of a gllvm-model describing community composition. The gllvm-models were fitted to presence–absence data from all 1500 sampling units (500 segments surveyed in 3 years). To separate the predictable and stochastic factors behind the four facets of community structure, we fitted an HMSC model where the four facets were considered as the response vector, and the predictors included abiotic environmental conditions (log mortality factor, as broken/uprooted/felled and diameter, decay class, ground contact and bark cover of the segment), a variable representing the identity of the colonizing individual (fungal strain, with control as a baseline, excluded for the year 2019) and a variable representing sampling effort (log-transformed number of sequence reads). As random effects, we included the log and site identities, again with the motivation that these capture latent log- and site-level covariates that have influenced all segments of the same log in the same way. We assumed linear models for the two latent variables, log-transformed DNA amount and log(*x* + 1) transformed OTU richness. We fitted the models separately for each year because the effects of both the fixed and random effects can differ during community development. We then decomposed the values of the four facets of community structure to the predicted value and the residual, which we viewed as the predictable and stochastic components of variation, respectively. We measured the amount of divergence for each segment by the log-transformed square of the residual. To ask how the level of divergence varied among the segment types and years, we performed an analysis of variance, where the divergence was the response variable, and the explanatory factors included the year (2019, 2020 and 2021), the log type (natural or felled), the treatment (control or inoculated) and the interactions between year and log type and year and treatment.

#### (iv) Model fitting

All analyses were performed in the R environment 4.3.1 [38]. For all models, we assumed the default priors of HMSC [36], which can be considered minimally informative (ch. 8 in [33]). We sampled the posterior distribution with four Markov chain Monte Carlo (MCMC) chains, each consisting of 37 500 iterations out of which the first 12 500 were considered as transient, and the remaining thinned by 100 to result in 250 samples per chain and hence 1000 samples in total. MCMC convergence was assessed through the potential scale reduction factors [39] of the estimated parameters. We confirmed that the models for facets of community divergence satisfied the assumptions of the underlying linear models, namely normality and homoscedasticity of residual variation (electronic supplementary material, appendix 5 figures S3 and S4).

## 3. Results

### (a) Stochasticity in colonization success

We detected *F. rosea* as DNA from 39% of the inoculated segments and as fruit body from 29% of the inoculated segments, more so from the felled than the naturally fallen logs (table 1). The control segments confirmed the causality of inoculations: we detected *F. rosea* as DNA only from one control segment in each of the years 2019−2021 (different segment each year) and as fruit body only in one year from one control segment. The experimental data revealed that the colonization success of *F. rosea* was highly predictable when measured as presence−absence, especially when measured from the fruit body rather than DNA data, but basically unpredictable when measured as DNA read count abundance (table 1). For both the fruit body and the DNA presence–absence, the highest predictability was reached for the pooled data that consisted of both naturally fallen and felled logs (table 1). This is expected, as the colonization rate varied between these two groups, and the model included log type as a predictor. Predictability was especially low for colonization success measured as fruit-body presence in natural logs, but as this group received only a small number of colonizations in total (*n* = 20, 10% of 200 sampling units), the cross-validation-based estimate of predictive power may not be very robust.

**Table 1.** Results of colonization success experiments. (For the colonization rate, the proportion of segments that became colonized in the sense of fruit-body presence or DNA presence is shown. For variance partitioning, the entries of the table show proportions of explained variance attributed to different groups of predictors. For predictive power, the entries of the table show 10-fold cross-validation based on Tjur’s *R*^2^ for fruit body and DNA presence–absence, and *R*^2^ for DNA abundance. The results are shown for each of the three response variables (fruit-body presence–absence, DNA presence–absence, DNA abundance) and for models fitted to each subset of the data (all, all segments; natural, natural segments only; felled, felled segments only). For proportions of explained variance attributed to the individual predictors rather than their grouping, see the electronic supplementary material, appendix 5 table S5. For posterior mean effects of the explanatory variables, see the electronic supplementary material, appendix 5 table S6.)

response variable	fruit-body presence–absence			DNA presence–absence			DNA abundance		
dataset	all	natural	felled	all	natural	felled	all	natural	felled
**colonization rate** (%)	29	10	48	39	24	55			
**variance partitioning**									
environment *(%)*	44	41	18	37	28	26	38	31	23
biotic *(%)*	6	23	8	20	40	14	31	30	44
individual *(%)*	15	21	19	6	8	22	16	22	21
sequencing depth *(%)*	0	2	1	1	1	1	2	3	2
random: site *(%)*	5	10	3	19	6	31	7	2	4
random: log *(%)*	30	21	52	17	16	6	7	12	6
**predictive power**	0.47	0.17	0.46	0.30	0.27	0.18	0.00	−0.03	0.00

The predictability of *F. rosea* colonization success was mostly explained by the log properties, i.e. type of the log, diameter, decay class, ground contact and bark cover of the segment (table 1). The biotic properties of resident fungal communities (i.e. DNA amount, OTU richness and community composition) prior to inoculation further contributed to explaining the colonization success of *F. rosea,* but more when measured as DNA presence–absence than fruit-body presence–absence (table 1). Conversely, the identity (strain) of colonizing individuals explained more variation in the fruit body presence–absence than in the DNA presence–absence (table 1). Sequencing depth explained almost no variation, suggesting that it was sufficient for DNA-based detection. The random effects of log and site explained a major part of *F. rosea* presence–absence, suggesting that there were other relevant abiotic and biotic log- and site-level predictors than those included in the model (table 1).

### (b) Apparent stochasticity in observational data

In contrast with the experimental results revealing a high predictability in colonization patterns of *F. rosea,* the observational occurrence data on *F. rosea* yielded highly unpredictable patterns. Based on 10-fold cross-validation, the amount of apparent stochasticity was very high in the models fitted to the observational data, especially when not considering the random effect of the study area. For the model without the random effect, predictability was close to zero (Tjur’s *R*^2^ = 0.03), and thus the amount of apparent stochasticity was close to one (0.97). For the model with the random effect, predictability increased threefold (Tjur’s *R*^2^ = 0.11), and thus the amount of apparent stochasticity decreased although it was still close to one (0.89). Furthermore, in the observational occurrence model, only 25% of the explained variation was attributed to log properties (log mortality factor 20%, log diameter 4% and decay class 1%), whereas the vast majority (75%) was attributed to the random effect of the site.

### (c) Stochasticity in community composition during succession

**Figure 2.**
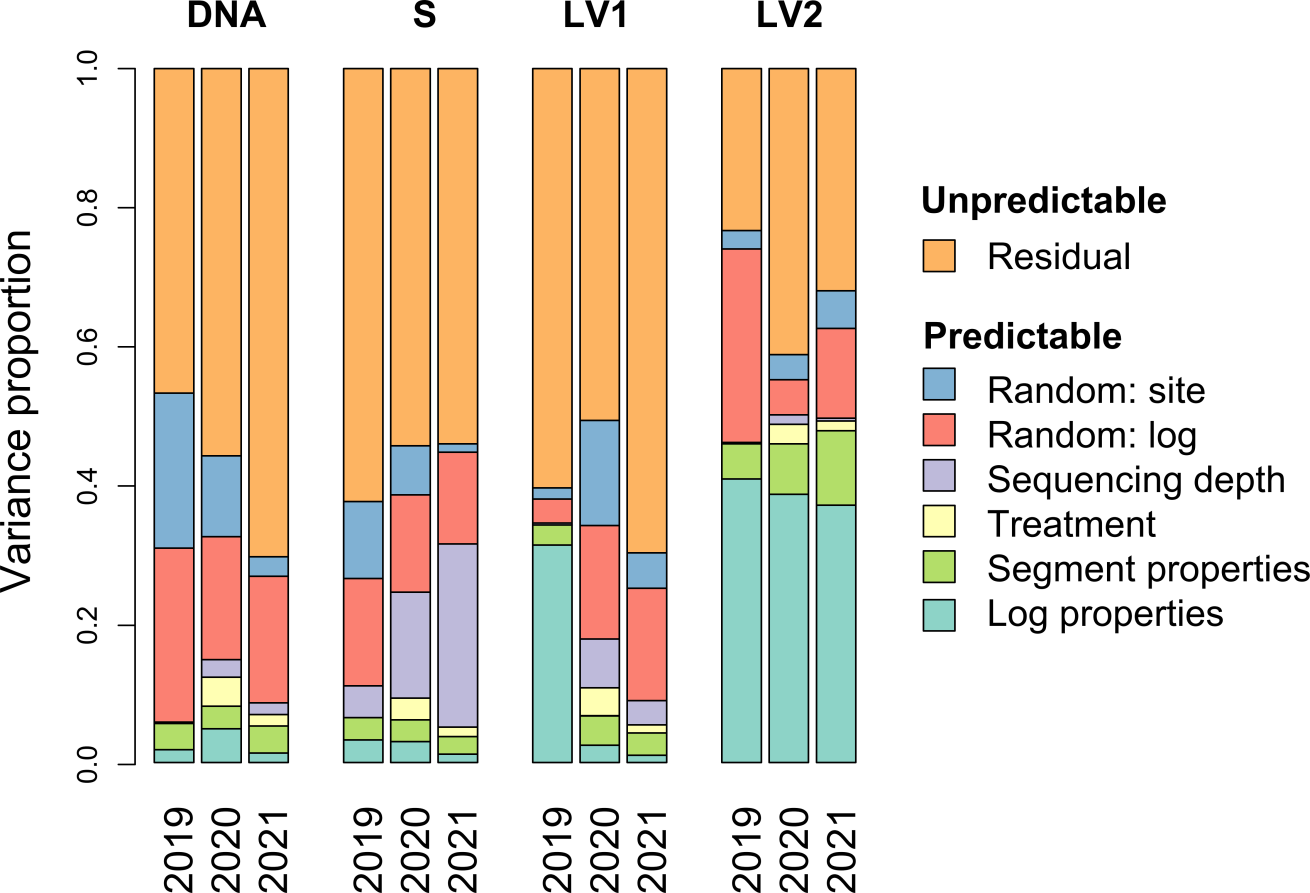
Predictable and unpredictable components of community succession. The bars show proportions of variance explained by different predictor groups, for each year of study (2019, 2020, 2021) and each community facet (DNA amount, species richness S, and community composition measured by the latent variables LV1 and LV2). Our main focus is on the residual variation interpreted here as community divergence owing to unpredictable stochastic variation, and which is further examined in table 2.

Despite conducting a highly replicated experiment, community succession was shown to be highly unpredictable in the sense that community succession diverged greatly across replicates. In quantitative terms, more than half of the variation in models targeting community divergence in DNA amount, species richness and fungal community composition remained unexplained (figure 2). The most predictable community facet was community composition measured by the second latent variable LV2, which could be predicted especially based on the log properties (figure 2). After controlling for the predictors, the remaining residual variance, which we interpret as stochastic divergence, showed statistically significant variation, especially among the years (table 2). From the points of view of DNA amount, species richness and latent variable LV1, the year 2020 (i.e. 1 year after the inoculations) had the highest amount of stochastic divergence (table 2). For DNA amount and species richness, divergence increased from 2019 to 2020 and then decreased from 2020 to 2021. For LV1, the years 2019 and 2020 did not differ, but then the divergence decreased from 2020 to 2021. For species richness, the interaction of log type and year was also significant, and the pairwise comparisons highlighted that natural logs experienced a higher level of divergence than felled logs in 2020 (table 2). Community composition measured by LV2 also showed a statistically significant interaction between log type and year, but this effect was mild in the sense that none of the pairwise comparisons were statistically significant (table 2).

**Table 2.** Results of analysis of variance in the facets of community divergence, including DNA amount, species richness and community composition measured with latent variables LV1 and LV2. (Statistical significances are coded as <0.001 (***), <0.01 (**), <0.05 (*) and >0.05 (). For statistically significant terms, we also show in *italics* Tukey multiple comparisons of means, shown for consecutive years for a year, and for significant comparisons for log type x year. Significances of the pairwise comparisons are coded as <0.001 (>>>), <0.01 (>>), <0.05 (>) and >0.05 (=).)

response	term	d.f.	sum of squares	Pr(>*F*)
DNA amount	log type	1	1.2	
	treatment	1	0.8	
	year	2	44.34	***
	*2019<<<2020*, *2020>>>2021*			
	log type x year	2	1.1	
	treatment x year	2	0.7	
	residual	1491	1435	
species richness	log type	1	1.5	
	treatment	1	0.0	
	year	2	128.3	***
	*2019<<<2020*, *2020>>>2021*			
	log type x year	2	13.9	***
	*F2020 << N2020*			
	treatment x year	2	0.0	
	residual	1491	1193.9	
LV 1	log type	1	3.0	
	treatment	1	1.3	
	year	2	14.4	**
	*2019 = 2020, 2020>>2021*			
	log type x year	2	1.3	
	treatment x year	2	3.2	
	residual	1491	2111.2	
LV 2	log type	1	0.0	
	treatment	1	0.2	
	year	2	2.8	
	log type x year	2	11.2	**
	*not significant (F2020 > N2020 with p = 0.07*)			
	treatment x year	2	0.3	
	residual	1491	1507.9	

## 4. Discussion

Our field experiment controlled for several ecological factors that cannot be controlled for in observational studies, allowing us to quantify parts of the variation in colonization and community succession representing true stochasticity and apparent stochasticity. The comparison between our experimental and observational data confirmed that the level of unpredictability of the observational data can be much reduced by controlled field experiments. Inoculations of the target species into comparable replicates allowed us to control for the stochasticity involved in the dispersal phase (e.g. stochasticity in the arrival time of dispersal propagules and the source strength), which is difficult to assess in observational studies. Additionally, inoculating different strains of the same species allowed us to measure the stochasticity in the colonization phase driven by the inter-individual differences in colonization capabilities, which we demonstrated to influence the colonization success of the focal species. Finally, having a high number of biotically and abiotically comparable replicates allowed us to reveal the high amount of stochasticity involved in fungal community succession.

Given that both the experimental and observational data included environmentally comparable logs, why was the amount of unpredictability in the observational data prominently higher than in the experimental data? We consider one main reason for this to be that the experimental and observational data encompass different phases of the fungal life cycle, and thus the results reflect that the relevance of stochasticity varies across the different life-cycle phases. The inoculants used in the experiment were formed by secondary (dikaryotic) mycelia and thus excluded the part of the fungal life cycle where haploid spores germinate and produce the primary (haploid) mycelia, and in which primary mycelia encounter each other to mate and form secondary mycelia. Hence, our results suggest that a major part of unpredictability in observational occurrence data relates to whether the dispersal propagules (i.e. haploid spores) are able to disperse to the focal log, germinate and find compatible haploid mycelia for mating, which would then yield the secondary mycelia used in the experimental study. In fact, compared with the primary mycelium, the secondary mycelium is superior in resource utilization [40]. Moreover, inoculum size, which was greater for our inoculated individuals than it would be under natural conditions, is known to enhance establishment success [41]. If these conditions hold, the experimental results suggest that the remaining part of the secondary mycelial colonization process and fruit-body formation are relatively deterministic. This interpretation is supported by the fact that the random effect of the site, which probably captures regional variation in fungal spore source strength, was a much more important predictor for the observational data than for the experimental data. The high proportion of variance attributed to the random effect means that it is especially difficult to make predictions about new study areas from which the prevalence of species is not known and hence, the random effect part of the model cannot be used for making predictions [42]. Furthermore, our results highlight that predicting which logs are and which are not occupied by *F. rosea* is very difficult also because many relevant predictors, such as the identity of colonizing individuals, will not be known in observational studies, generating patterns of apparent stochasticity.

However, we note that even if the experimental study was directly targeted to separate the effect of stochasticity, doing so is very challenging, as it is not straightforward even to conceptually define what types of variation should be considered stochastic versus deterministic. In our experiment, despite aiming for as similar initial conditions as possible, the different segments of the same log were likely to vary in their abiotic and biotic conditions. We aimed to control for this variation through the covariates included in the models, but some deterministic variation admittedly remained uncontrolled for. Furthermore, we note that it was challenging to make the comparison between observational and experimental logs completely fair. For instance, the cut-end experimental logs were directly exposed to the environment and provided colonization gateways for fungi. As another example, while we included different strains of the focal species in the experiment to mimic natural variability, it is very difficult to evaluate how much genetic variability there is in colonization attempts, including the failed ones. While it seems difficult to overcome such limitations, we still consider it informative to compare the levels of stochasticity observed in a field experiment to those observed in natural occurrences.

The colonization success of *F. rosea* was explained by the deterministic extrinsic factors related to the abiotic log properties and the composition of resident fungal communities, and by the intrinsic factors related to the identity of colonizing individuals. The influence of log properties on wood-inhabiting fungal occurrences has repeatedly been reported [43–45]. More interestingly, we found the biotic conditions of the logs prior to inoculation to be a relevant predictor for *F. rosea’s* colonization success, but more for mycelial establishment (and thus for detection as DNA) than for fruit-body production. Namely, confirming previous results from small-scale experiments, interactions with the resident species seem to play an important role in determining the colonization success of wood-inhabiting fungi [46,47]. On the other hand, our results indicated that strains of the colonizing individuals especially influenced their capacity to produce a fruit body. This is in line with the idea that different fungal genotypes are adapted to different environmental conditions and that the investment of individuals in sexual reproduction depends on such a level of adaptation [48].

Our results showed that stochasticity is more prominent in the initial phase of community succession, with the earliest successional stage involving a higher level of stochasticity than the later successional stages. This result is consistent with earlier studies showing that stochastic divergence plays a major role in early community succession, but at later stages, community structure stabilizes [19,20]. Following the framework by Dini-Andreote *et al*. [19], the shifts in the balance of stochastic and deterministic processes during microbial community succession arise from selective processes that intensify as the succession progresses. According to this framework, early colonizers are generalists and weak competitors, leading to weak selection and strong stochasticity. However, as succession proceeds and organisms affect their environment (e.g. through resource utilization), selective processes of abiotic habitat filtering and biotic competition (*sensu* [1]) become more important. Even if our study considered only the initial stages of wood decomposition, it captured such patterns. However, we note that our data contained only 3 years, and thus uncontrolled differences in, e.g. climatic conditions between the years may also have contributed to the observed patterns. Furthermore, longer term data would be necessary to assess whether deterministic processes stabilize at later phases of species succession. Our results also showed that community divergence was greater for natural than for felled logs, the former of which provides a wider range of microhabitats and hosts more diverse fungal communities [23]. This is in line with earlier literature where the importance of stochasticity has been found to be higher in more complex biotic and abiotic environments [6–8]. This finding suggests priority effects can lead to multiple community transient states (*sensu* [49]).

By surveying the colonization success of the focal species through morphological identification and molecular methods simultaneously, we demonstrated that the species observation method strongly influences the estimation of stochasticity in fungal communities. The colonization success of *F. rosea* was highly predictable when measured as presence–absence, especially when measured from fruit-body observations rather than from DNA surveys, but basically unpredictable when measured as DNA read count abundance. Fruit-body surveys are prone to false negatives owing to missing individuals present only at the mycelial stage [50]. However, unlike many other fungal species, *F. rosea* produces conspicuous fruit bodies quickly after mycelial colonization [37], and the fruit-body surveys were based on careful examination of the entire segments. By contrast, DNA-based surveys are prone to many kinds of false negatives and false positives. To start with, they are based on a tiny, localized sample of sawdust, in which the focal species may be missing even if it is present elsewhere in the segment, probably leading to a substantial proportion of false negatives. Furthermore, DNA barcoding involves stochasticity in the sequencing procedure, such as PCR stochasticity [51], and primer amplification biases [52]. While DNA read counts and relative abundances generally correlate [53], using sequence reads from a small subsample as a quantitative measure of abundance is challenging, and it may yield inflated estimates of apparent stochasticity. These challenges are especially important to take into account when estimating apparent stochasticity in microbial communities characterized through DNA-based surveys, for which there has been an increasing interest in the recent literature [13,15–17,19]. While the species assignment phase also generally involves a lot of uncertainty owing to the incompleteness of reference libraries [31], in the present case, we only aimed to detect the presence of one species that was well covered in the reference data, and hence we do not consider misclassification to contribute much to the recorded level of apparent stochasticity.

## 5. Conclusion

The effect of stochasticity in community assembly has been much studied with the help of modelling and highly controlled laboratory conditions, but it has remained difficult to study under natural conditions. Our results show that the amount of true stochasticity in fungal colonization is much less than could be expected from the apparent stochasticity recorded from observational studies. The comparison between our experimental and observational results suggests that stochasticity in dispersal and the initial establishment phase plays a major role in generating stochasticity in fungal occurrence patterns, whereas there is less stochasticity in the subsequent resource acquisition and fruit-body production. Our results further confirm the earlier expectation that the initial phase of community succession involves a higher level of stochasticity than the later stages of community succession. We conclude that measuring the amount of stochasticity in core community assembly processes is feasible for species-rich communities under natural conditions and highlight the importance of testing the generality of our results in other types of systems. We hope future studies test the generality of our results also with focal species other than *F. rosea* and assess the role of stochasticity in long-term data.

## Data Availability

Raw sequences are deposited to the European Nucleotide Archive: https://www.ebi.ac.uk/ena/browser/view/PRJEB65750. The data and scripts needed to reproduce the analyses are provided at Zenodo [54]. Supplementary material is available online [55].
